# Diffusion Behavior of Polyethylene Furanoate (PEF) and Tritan as Sustainable Polyester Packaging Materials

**DOI:** 10.3390/polym17192674

**Published:** 2025-10-02

**Authors:** Frank Welle

**Affiliations:** Fraunhofer Institute for Process Engineering and Packaging (IVV), Giggenhauser Straße 35, 85354 Freising, Germany; frank.welle@ivv.fraunhofer.de

**Keywords:** PEF, Tritan, polyester, sustainable polymers, barrier properties, diffusion coefficients

## Abstract

Polyethylene furanoate (PEF) and Tritan^TM^ copolyester are sustainable polyester polymers. PEF is made from biobased resources, whereas Tritan is mainly used for reusable food contact articles. Both polyesters are alternatives for polyethylene terephthalate (PET), which is currently the most used polyester in food packaging. Like all packaging polymers, sustainable alternatives to fossil-based PET must also comply with food law requirements. Prediction of the migration can be used as an alternative to complex and time-consuming experimental migration measurements. Since there are no such predictive models for either PEF or Tritan, the modelling parameters for PEF and Tritan were determined in this study from experimentally determined diffusion coefficients and activation energies. The diffusivity of PEF and Tritan was compared with PET and polyethylene naphthalate (PEN). Of the four polyester polymers, PEF shows the lowest diffusion, followed by PEN, PET, and Tritan. Overall, the results show that the investigated polyesters are low-diffusivity polymers.

## 1. Introduction

Polyethylene terephthalate (PET) is used widely as a packaging material for beverages like mineral water, soft drinks, and juices. PET is a clear, transparent polymer that can also be 100% recycled [1]. Collection systems and recycling processes for PET bottles have been established all over the world. Moreover, most of them are already categorized as “suitable technologies” according to European Regulation 2022/1616 [2]. These suitable technologies can be used in Europe to recycle PET bottles and trays and are considered safe by the European Commission, the European Food Safety Authority (EFSA), and National Competent Authorities. However, PET is made from fossil-based resources, which is considered a disadvantage. In principle, PET, which is polymerized from the monomers ethylene glycol and terephthalic acid (or its dimethyl ester), can also be produced from renewable raw materials [1]. However, to date, this bio-based version of PET, a so-called plug-in polymer, has not yet reached a significant market share. While ethylene glycol is available from sugar cane and the ethanol produced from it, the second monomer, terephthalic acid, is very difficult to produce from renewable raw materials. As a result, the bio-based proportion is usually only around 30%, i.e., the mass fraction of ethylene glycol in the total PET polymer.

An alternative for PET made entirely from renewable raw materials, polyethylene furanoate (PEF), is about to be launched on the market. PEF also uses (bio-based) ethylene glycol as a monomer, but instead of terephthalic acid or its dimethyl ester, it uses furane dicarboxylic acid (FDCA) or its dimethyl ester produced from biobased glucose as a second monomer. PEF is therefore 100% bio-based and has similar mechanical properties compared to PET. PEF is therefore a sustainable alternative to the widely used PET in the beverage packaging sector. It is to be expected that PEF will be used in similar areas of packaging as PET [3,4,5]. The better barrier properties of PEF compared to PET may even make further applications possible. For example, PEF has a barrier for CO_2_, which is 19 times better compared to PET [6]. The oxygen barrier is 10 times better than for PET [7].

Another polyester in the packaging sector is Tritan^TM^ copolyester. Tritan is polymerized from the monomers dimethyl terephthalate (DMT), 1,4-cyclohexane dimethanol (CHDM), and 2,2,4,4-tetramethyl cyclobutane-1,3-diol (TMCBD). Although the polymer is not bio-based like PEF, it is very often used for reusable applications, e.g., in reusable bowls, drinking cups and other catering applications. Its significantly better temperature resistance compared to PET allows it to be hot-washed after use and is then available again for the next filling. Tritan can therefore also be seen as a sustainable alternative to PET used for reusable items, even if Tritan’s market share is much lower than that of PET. In European legislation, great importance is attached to ensuring that the proportion of reusable applications is significantly increased [8]. This will most probably also lead to a higher market share for Tritan in the near future.

Like all packaging polymers, sustainable alternatives to fossil-based PET must comply with food law requirements. In particular, packaging materials must comply with the more general requirements of the European Framework Regulation 1935/2004 [9] and fulfil the specific requirements of European Regulation 10/2011 [10]. Comprehensive analyses of the migration of monomers, additives, and processing aids, but also of non-intentionally added substances (NIAS), are required for this, as well as testing of the organoleptic properties of the packaging material. These tests are necessary but also cost-intensive and time-consuming. Finally, the distributor of the packaging must provide a comprehensive “Declaration of Compliance” for its packaging polymer and thus demonstrate that all regulations and specific migration limit values are complied with. Compliance with the specific migration limits of monomers and additives is more a routine test and comparatively easy to carry out using validated methods for the substances. In the case of NIAS, it is much more difficult to prove compliance with the current regulations. NIAS are by-products from polymerization such as oligomers, sporadic impurities, degradation products of additives, etc. These substances usually have low and fluctuating concentrations, which often makes NIAS difficult to analyze and assess according to food law compliance. In the case of reusable applications, substances from the first use, e.g., flavour compounds, are also considered as NIAS. These can diffuse into the polymer in the first use, and then, if they are not completely removed during washing, migrate into the food during repeated filling.

While intentionally added substances (IAS) such as monomers, additives, and processing aids generally have defined and usually comparatively high specific migration limits, the requirements for NIAS are much more challenging. IAS are known and very well analyzed toxicologically. This enables categorization and relatively high specific migration limits, as critical substances can already be excluded in the production of the polymer. NIAS, on the other hand, are less well analyzed and therefore, in the interest of preventive consumer protection, also have significantly lower specific migration limits. For the oligomers of PEF, for example, EFSA has stipulated that the migration of oligomers from PEF less than 1000 g/mol should not exceed a concentration of 50 µg/kg (expressed as FDCA) [11]. This 50 µg/kg should not be understood as a specific migration limit for PEF oligomers; rather, it is the precautionary generic limit according to the Threshold of Toxicological Concern (TTC) concept if no specific data is available [12,13]. There is no such restriction on oligomers for Tritan [14], but due to the analogue chemistry of polyesters, the oligomers should also be evaluated [15]. A low migration limit of 50 µg/kg for PEF oligomers in food or food simulants is difficult to confirm because it is a cumulative limit value and the number and concentration of the individual oligomers can vary due to the polymerization conditions. In addition, quantitative analysis of NIAS in the low µg/kg concentration range is extremely difficult. This represents a particular hurdle for the two sustainable alternatives to PET.

Diffusion modelling can be used as an alternative to complex experimental migration measurements, especially for the evaluation of polyester oligomers [16] or other NIAS [17]. Diffusion modelling is also applied for the safety evaluation of recycled PET in food contact materials [18,19]. Modelling of the diffusion and migration is possible if the diffusion coefficients D_P_ of a migrant and the activation energies of diffusion E_A_ are available. For most substances, however, these diffusion coefficients and activation energies have never been determined experimentally. Therefore, the diffusion coefficients are estimated using prediction models, for example, from the molecular weight of the migrating substances or from its molecular volume [20,21,22]. In principle, this also enables the prediction of unknown migrants if the molecular weight or the molecular volume can be estimated, e.g., from the mass spectrometric measurements or from retention times of the gas chromatographic analysis. However, a basic pre-requisite for the modelling of migration is suitable parameters for the prediction of the diffusion coefficients of the migrants in the polymers. In the early days of migration modelling, it was common practice to use very over-estimated parameters in order to obtain sufficient safety factors against exceeding the specific migration limits. This led to very conservative modelling parameters that highly over-estimated the predicted diffusion coefficients and therefore also migration. Such conservative parameters may be useful if relatively high limit values apply and can be confirmed using conservative calculations. Low migration limits, however, can easily be exceeded simply because over-estimated diffusion coefficients are used for the calculation. Therefore, for more realistic migration modelling, only slightly over-estimated modelling parameters should be available. Also, the influence of temperature on migration should also be realistically modelled. This is made possible by determining the activation energy of diffusion E_A_ and using this to predict the diffusion coefficients D_P_. In general, the partition coefficient is also necessary to predict migration into the food. However, for low diffusivity polymers such as polyesters, the partition coefficient is not necessary because in typical applications the equilibrium between polymer and food is not reached.

A special case of food law assessment is the migration assessment of reusable packaging. The legal requirements stipulate that the third contact with the food is used to assess the specific migration values [10]. The third value has also to be compared with the first and second value. This can be easily achieved experimentally with three consecutive migration measurements. However, if migration modelling is used for the assessment, the modelling parameters must not be over-estimated. If the first and second migration contacts are over-estimated, the third contact, which is then used for the evaluation, will inevitably be too low. This leads to an under-estimation of real migration and a possible false positive evaluation of the reusable packaging. It is therefore essential that realistic and not over-estimated modelling parameters are available.

The aim of this study was the determination of realistic—i.e., not over-estimated—diffusion coefficients and the activation energies of diffusion in PEF and Tritan. For this purpose, diffusion coefficients were determined without interactions with food simulations using gas-phase migration kinetics. The activation energies of diffusion E_A_ were calculated from the temperature dependence of the diffusion coefficients.

## 2. Materials and Methods

### 2.1. Film Samples

Two films were used for the tests. These were provided by the manufacturing companies of the polyester materials:

- 14 µm biaxially oriented PEF film (provided by Avantium, Amsterdam, The Netherlands).
- 51 µm Tritan^TM^ film (provided by Eastman, Kingsport TN, USA).

### 2.2. Determination of Diffusion Coefficients

The diffusion coefficients of the organic substances in PEF and Tritan listed in Tables S1 and S2 (Supplementary Materials) were determined from permeation measurements according to the lag time method. The substances were selected to obtain a wide range of functional groups, polarities, and molecular weights. The molecular weights and volumes are also given in Tables S1 and S2. The substances were mixed and measured in several sets, e.g., all n-alkanes together (except the gaseous alkanes, which were measured separately), all alcohols, all ketones, all flavour substances, etc. So, each set contains between 4 (gaseous alkanes) and 13 (flavour substances) individual substances, which were measured at the same time.

The films are clamped in a measuring cell. The surface area of the film is 191 cm^2^. The measuring cell consists of a lower part with a large container (volume of 7667 cm^3^) that is doped with the permeants of interest. The upper part of the measuring cell is constantly flushed with nitrogen. The nitrogen stream carries the substances being permeated through the film and brings them to the enrichment unit where the permeants were trapped and concentrated over a period of 20 min. The enrichment unit was connected to a gas chromatograph with flame ionization detection (GC/FID), and the permeants were directly desorbed into the gas chromatograph. Using this method, one kinetic point per substance could be measured every 40 min. The measurements were carried out until the equilibrium state (constant permeation over time) was reached. The lag time can then be determined from the kinetic curve. Calibration was performed with injections of known amounts of the applied permeants. The diffusion coefficients (D_P_) were determined from the lag time and the film thickness l according to Equation (1) [23]. The activation energies of diffusion E_A_ and the pre-exponential factor D_0_ were calculated from the D_P_ at various temperatures according to the Arrhenius Equation (Equation (2)). As the thickness of the films is included in the measurements here, these were determined experimentally. For both polymers, the activation energies were only calculated if at least four diffusion coefficients and a temperature difference of at least 15 °C, with a minimum of four kinetic points, were given; otherwise, the activation energies of diffusion are too uncertain.

(1) $$lag\;time=\frac{l^{2}}{6D_{P}}$$

(2) $$D_{P}=D_{0}\;e^{-\frac{E_{A}}{RT}}$$

### 2.3. Diffusion Modelling

Diffusion modelling was performed with AKTS SML software version 4.54 (AKTS AG, Siders, Switzerland) using finite element analysis [24]. The calculations were carried out with the following boundary conditions: The layer thickness was assumed to be 300 µm but does not play a role in migration due to the low diffusivity of the four polymer esters. The partition coefficient was set to K = 1, which corresponds to good solubility of the migrant in the contact medium. The surface/volume ratio was set to 6 dm^2^/kg food (EU cube). The densities of the polyesters used for the calculations were set to 1.43 g/cm^3^ (PEF), 1.36 g/cm^3^ (PEN), 1.40 g/cm^3^ (PET), and 1.18 g/cm^3^ (Tritan).

## 3. Results

The diffusion coefficients of PEF and Tritan were obtained from permeation measurements. For both polymers, permeation of the organic substances followed Fick’s law so that the diffusion coefficients could be calculated from the lag time of the experimental permeation curves. Overall, 151 diffusion coefficients were determined for PEF from the lag time tests, with 39 organic substances with various functional groups, molecular sizes, and polarities. In addition, a total of 21 activation energies of diffusion E_A_ could be calculated from the temperature dependence of the diffusion coefficients in the correlation between the logarithm of D_P_ versus reciprocal temperature (Arrhenius plot). For Tritan, a total of 122 different diffusion coefficients were determined, with 37 organic substances, and overall, 20 E_A_ were calculated for Tritan. The results are shown in Table 1 (PEF) and Table 2 (Tritan). The complete results of the diffusion coefficients and activation energies are given in the Supplementary Materials in Table S1 (PEF) and Table S2 (Tritan). The temperature ranges and the number of kinetic points in the Arrhenius plot are also given in Table 1 and Table 2. Due to the low diffusion of the two polyesters, the temperatures had to be set very high. Using thinner films, which would have been an alternative to accelerate permeation, was not possible. A film thickness of 14 µm for PEF and a 50.6 µm thick film for Tritan are the thinnest films available for the two polyesters. It is important to note that PEF was measured above the glass transition temperature of 86 °C, whereas Tritan was measured below its glass transition temperature (110 °C). Due to the low diffusivity of PEF, the temperature has to be increased in order to measure the lag time in a reasonable time. Lag times below the glass transition temperature (e.g., at 70 °C) are in the range of several years and cannot be measured with the applied method.

## 4. Discussion

### 4.1. Diffusion Modelling Parameters

**Figure 1:**
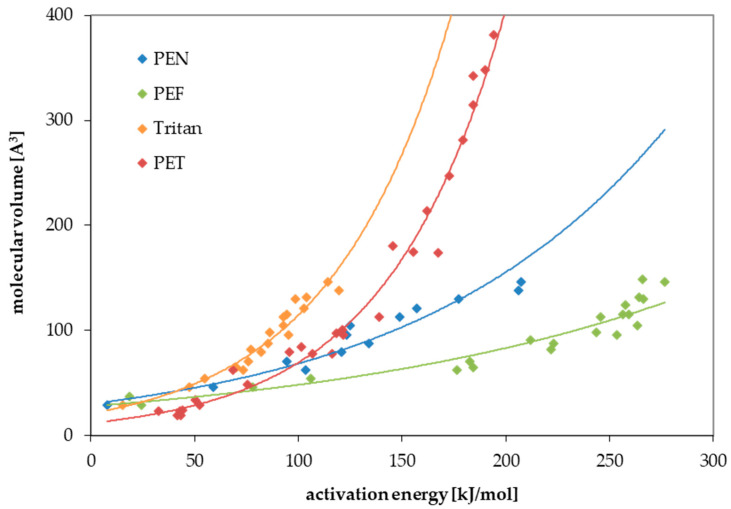
Correlation between the molecular volume V and the activation energies of diffusion E_A_ for PEF (this study), Tritan (this study), PEN [25], and PET [22].

**Figure 2:**
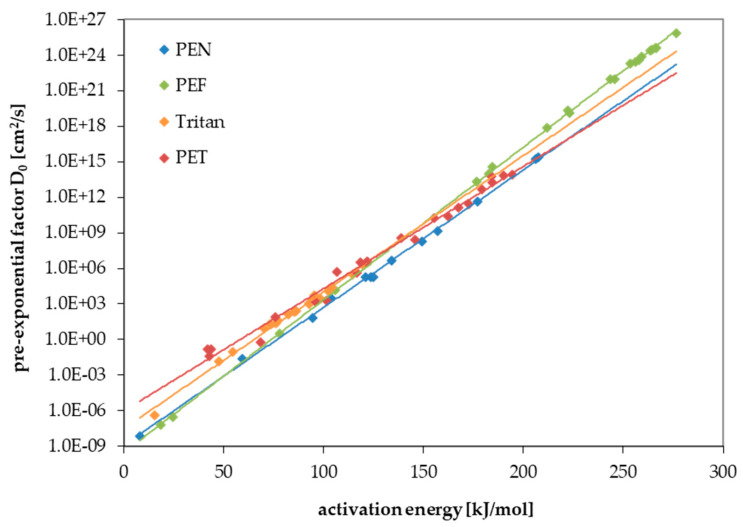
Correlation between the pre-exponential factor D_0_ and the activation energies of diffusion E_A_ for PEF (this study), Tritan (this study), PEN [25], and PET [22].

For PEF and Tritan, the diffusion coefficients and activation energies of diffusion were determined experimentally as part of this study. All permeation experiments followed Fick’s law of diffusion. This makes it possible to predict the diffusion of other substances that are not tested in this study. As already known from a previous work on PET [22] and polyethylene naphthalate (PEN) [25], two correlations are evident for the modelling parameters for the prediction of diffusion coefficients. First, E_A_ correlates with the size of the molecule. Larger molecules, therefore, have higher activation energies of diffusion than smaller molecules. For the correlation, the molecular volume V was used, which was calculated with the programme “Molinspiration” [26]. Similar correlations are also available with the molecular weight of the substances. The second correlation results for the pre-exponential factor D_0_ and the activation energy E_A_. This means that substances with low activation energies have small pre-exponential factors and vice versa. This is known for many activation energy-based processes (see, for example, [27,28]). Both correlations are shown in Figure 1 and Figure 2. For comparison, data also published for PET [22] and PEN [25] are included in the graphs. The increase in activation energies with increasing molecule size is strongest for PEF, followed by PEN, PET, and Tritan, which means that the same molecule has a much higher activation energy in PEF compared to Tritan. From the above-mentioned correlations, parameters can be derived which can be used to predict the diffusion coefficients for both polyesters. The intercepts and the slopes of the two correlations are used for this purpose. These are used as parameters a to d in Equation (3). This equation was derived in an earlier study from the above-mentioned correlations and the Arrhenius equation [22]. Parameters a to d are listed for PEF and Tritan in Table 3.

**Figure 3:**
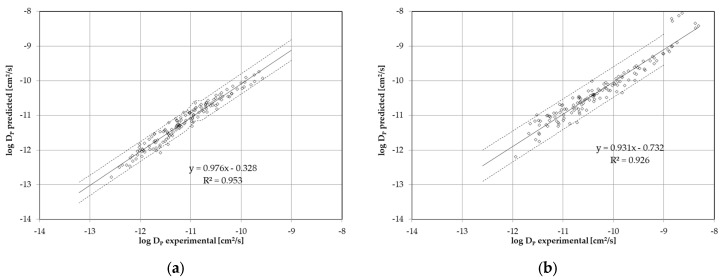
The log-log plot of the experimental and predicted diffusion coefficients: (**a**) PEF; (**b**) Tritan.

To test the quality of the prediction model of the diffusion coefficients, the predicted diffusion coefficients were compared with those determined experimentally in this study. The comparisons are plotted in so-called log-log plots (Figure 3). As a result, the slope for both correlations is almost 1, and both correlations go almost through the origin. This indicates a good prediction of the diffusion coefficients of the two polyesters. The dashed lines in Figure 3 determine the 95% confidence interval.

(3) $$D_{P}=b{\left(\frac{V}{c}\right)}^{\frac{a-\frac{1}{T}}{d}}$$

### 4.2. Literature Data for the Migration from PEF and Tritan

In order to compare the prediction of the diffusion coefficients and thus the possible migration of organic substances, i.e., the transfer of substances from the polymer into a contact medium of both polymers, the scientific literature was investigated. For Tritan there are few studies that deal with migration. Migration studies on PEF for oligomers or other NIAS are to the knowledge of the author not available in the scientific literature. The reason might be that PEF is currently starting to enter the market; therefore, only a limited number of materials and food contact items are available for migration studies. On the other hand, information about the oligomeric fraction is available for PEF. Hoppe et al. [29] found 20 oligomers overall (in four groups with individual monomers) in PEF. Group I (EG and FDCA) represents the mass of 87% of all detected oligomers, whereas group II (EG, DEG and FDCA) represents 12%, with groups III and IV representing 1%. Unfortunately, concentrations of the oligomers in PEF were not given in this study.

Regarding Tritan, several studies have been published on the migration of sub-stances from Tritan into food simulants. The migration of bisphenol A (BPA) from Tritan into water for 120 h at room temperature was studied by Cooper et al. [30]. BPA is not used in the production of Tritan and, therefore, it is not surprising that the migration of BPA could not be detected at a detection limit of 0.01 µg/L. The migration from Tritan into 50% ethanol at 70 °C for a storage time of 2 h was studied by Simoneau et al. [31]. Even if the temperature is much higher than in the study of Cooper et al., and 50% ethanol might swell the surface of the Tritan bottle, the migration was below the detection limit of the applied method of approx. 1 µg/L. Guart et al. [32] measured the migration from Tritan into water for storage of 10 days at 40 °C. Several substances (possibly contaminants), including BPA, benzyl butyl phthalate, 2-phenoxyethanol, and dimethyl *iso*-phthalate, were detected in concentrations of up to 1 µg/L in the migration solutions. In another migration study by Onghena et al. [33], a Tritan baby bottle was investigated at 70 °C for a storage time of 2 h. In contrast to the findings of Simoneau et al. [31], Onghena et al. [33] found a large amounts of migrants in the applied simulants. However, the detected essential oil compounds (camphor, eucalyptol), 2,6-di-*iso*-propyl naphthalene, and benzophenone are more related to contamination, e.g., from recycled paper/board, than to the Tritan polymer. The highest concentration was found for 2,6-di-*iso*-propyl naphthalene, with 30 µg/L. Unfortunately, in all migration studies [30,31,32,33], the concentrations of the migrants in the Tritan material were not determined. This makes it impossible to determine whether the above-mentioned migration values were caused by diffusion of the substances from Tritan or by contamination. Also, migration cannot be predicted if the concentration of the substances is not known in the polymer.

In the most recent migration study by Kubicova et al., the oligomeric fraction was determined to be approx. 1.4 wt% as a sum of all oligomers [34]. The variation in the oligomeric structures found in Tritan is much higher than for other polyesters because Tritan is manufactured from three monomers (dimethyl terephthalate, CHDM, and TMCBD), and both diols have two diastereomers each [35]. This increases the variety of different oligomeric structures, which might be the reason why the individual oligomeric structures are not given in the scientific literature. In addition, due to the lack of commercially available reference oligomers, quantitative information on oligomers is lacking.

Kubicova et al. [34] also investigated the migration of Tritan oligomers into food simulants at storage conditions of 10 days at 40 °C (3% acetic acid, 20% ethanol, 50% ethanol), 2 h at 70 °C (3% acetic acid, 20% ethanol, 50% ethanol), 30 min at 121 °C (sunflower oil), and 2 h at 100 °C (3% acetic acid, 10% ethanol). As a result, the migration of the sum of oligomers at 40 °C storage conditions was below the analytical detection limit of 25 µg/L. Only at high temperatures of 100 °C or with swelling simulants (50% ethanol) concentrations above the detection limits were found for the sum of Tritan oligomers, e.g., 170 µg/L (2 h at 70 °C, 50% ethanol), 379 µg/L (30 min at 121 °C, sunflower oil), 108 µg/L (2 h at 100 °C, 10% ethanol), and 33 µg/L (2 h at 100 °C, 3% acetic acid).

In summary, it can be concluded that the migration of substances from Tritan is very low and is presumably in the lower µg/L range for individual substances. However, as the individual oligomers were not quantified, no migration prediction for individual substances can be made here.

### 4.3. Prediction of Maximum Concentrations of Oligomers and Other NIAS

As mentioned above, migration studies into food simulants were published for PEF and Tritan but without determination of the corresponding concentrations in the polymers. Concentrations of the migrants in the polymer, however, are necessary to calculate the migration using migration modelling. Therefore, it is not possible to predict the migration with the actual modelling parameters for PEF and Tritan and compare them with the published experimental migration studies. Since quantitative data on concentrations of individual oligomers and other NIAS are not available for PEF and Tritan, the concentrations in the polymer were calculated, which correlate with migration limits, e.g., 10 µg/L as a typical limit for NIAS. This makes it possible to assess compliance with existing limit values by quantitatively determining the concentrations in the polymers. The migration was calculated using the modelling parameters determined in this study (Table 3). For given migration conditions, e.g., 365 days storage time at 25 °C and 1 kg food packed in 6 dm^2^, the migration is directly proportional to the concentration in the polymer. This means that if a concentration in the polymer correlates with a migration limit of 10 µg/L, then, for example, the 5-fold concentration in the polymer correlates with a limit of 50 µg/L. This makes the calculations in this study universally applicable to any specific migration limit.

Migration was predicted from the modelling parameters determined in this study for the following conditions:

- Storage for 365 days at 25 °C (long-term storage conditions, e.g., for mineral water bottles as applied by EFSA for the evaluation of recyclate containing PET bottles [19]).
- Storage for 10 days at 60 °C as official testing conditions for long-term storage at room temperature according to Regulation 10/2011 [10].
- Heating to 70 °C for 2 h to simulate hot fill conditions.
- Heating to 100 °C for 30 min to simulate microwave treatment.

**Figure 4:**
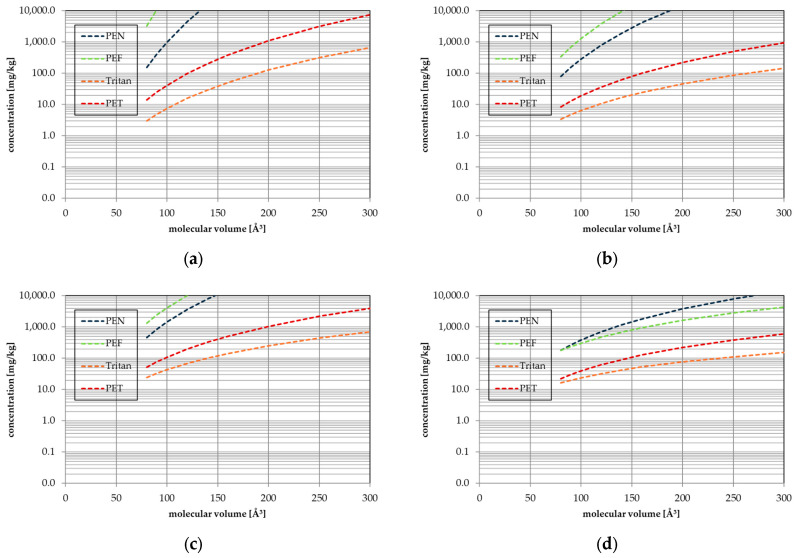
Concentrations of migrants in the polymer corresponding with a migration of 10 µg/L: (**a**) 365 days at 25 °C; (**b**) 10 days at 60 °C; (**c**) 2 h at 70 °C; (**d**) 30 min at 100 °C.

In addition to PEF and Tritan, the polymers PET and PEN were also calculated for comparison. The concentrations of migrants up to 300 Å^3^ in the polymer corresponding with a migration of 10 µg/L after storage for (a) 365 days at 25 °C, (b) 10 days at 60 °C, (c) 2 h at 70 °C, and (d) 30 min at 100 °C are given in Figure 4. As a result, under moderate-temperature conditions, PEF is the lowest diffusive polymer of the four investigated polyesters, followed by PEN, PET, and Tritan. Tritan is an amorphous polymer, whereas PEF, PEN and PET are partly crystalline, which explains the higher diffusivity of Tritan. Going to higher temperatures, however, the difference in diffusivity of the four polyesters becomes smaller. This is due to the higher activation energies of PEF, PEN, and PET compared to Tritan (Figure 1). A higher activation energy for a particular molecule means that the influence of temperature has a greater effect on the increase in the diffusion coefficient with increasing temperature.

The four polyesters investigated behave very differently, which will be illustrated using the example of a molecule with a molecular volume of 100 Å^3^ (e.g., the solvent toluene). The concentrations corresponding with a migration of 10 µg/L after storage for 365 days at 25 °C are 30,000 mg/kg for PEF, 989 mg/kg for PEN, 41.2 mg/kg for PET, and 7.47 mg/kg for Tritan. The concentration of this molecule in PEF can therefore be around 4000 times higher than in Tritan with the same migration value. At a temperature of 60 °C, the differences are smaller. The concentrations corresponding with a migration of 10 µg/L after storage for 10 days at 60 °C are 1280 mg/kg for PEF, 282 mg/kg for PEN, 18.9 mg/kg for PET, and 6.41 mg/kg for Tritan. The difference between PEF and Tritan is therefore only a factor of about 200. At 100 °C, even PEN is the lowest diffusive polyester instead of PEF. A migration of 10 µg/L after storage for 30 min at 100 °C allows for 305 mg/kg in PEF, 378 mg/kg in PEN, 39.2 mg/kg for PET, and 23.8 mg/kg for Tritan, respectively. The difference between PEF and Tritan is, at 100 °C, a factor of about 13. A molecule with 300 Å^3^ (e.g., *n*-octadecane) allows for concentrations of 4350 mg/kg for PEF, 14,800 mg/kg for PEN, 611 mg/kg for PET, and 154 mg/kg for Tritan, which correspond to a migration of 10 µg/L after storage for 30 min at 100 °C. This results in a factor of about 28 between PEF and Tritan. The results show that understanding diffusion in polymers is very important for compliance testing. Even if only one class of polymers, e.g., the polyester family PEF, PEN, PET, and Tritan, is considered, the effects on the diffusion, and therefore on migration and consumer exposure, are enormous. The results show also that the activation energy of diffusion plays an important role when evaluating the migration of substances from the polymer into contact media.

At this point it also becomes obvious that the concentrations in the polymer can be very high, even at high temperatures such as 100 °C. All NIAS are typically not present in such high concentrations, and intentionally added substances such as additives are certainly not. The highest concentrations of NIAS in polyester are the oligomers. The smallest oligomer for PEF is the cyclic dimer, with a molecular volume of 287 Å^3^. At temperatures below 70 °C, the concentration in the polymer can be well above 10,000 mg/kg without exceeding the migration of 50 µg/L. However, this limit applies to the sum of all oligomers. This is probably difficult to determine experimentally in migration tests because the concentrations and the cumulative migration limit value are very low. The approach chosen in this study can be used for compliance assessment. Determining the concentrations of the oligomers in the polymers is much easier and faster than determining the migration into food (simulants). Using the modelling parameters determined in this study, the upper limit of oligomers in the polymer can be defined, which correlate with a certain limit value (e.g., 50 µg/L).

Tritan is the highest diffusive polymer compared to PEF, PEN, and PET. However, Tritan is used in most cases in short-term contact applications. The results of this study show that concentrations of NIAS in the material above 10 mg/kg for very small molecules like solvents and above 100 mg/kg are acceptable without exceeding the migration limit of 10 µg/L. In addition, Tritan is used for reuse applications. Articles are hot-washed before use and before each refill. As diffusion is a reversible process, NIAS or substances that have migrated from the application (e.g., flavour substances) are removed again during washing.

## 5. Conclusions

As part of the study, the parameters for predicting diffusion were determined for two polyesters, namely, PEF and Tritan. These now make it possible to predict the migration of NIAS, oligomers, or other substances from the polymers into the food. Predicting migration can simplify the compliance evaluation of food contact items made from PEF or Tritan. However, due to the low diffusivity of PEF, the diffusion coefficients of PEF had to be determined at higher temperatures between 90 °C and 120 °C. This is above the glass transition temperature of PEF. Typical applications of PEF are at ambient temperatures. The extrapolation of the diffusion coefficients therefore takes place over a very wide temperature range, which increases the uncertainty. On the other hand, diffusion above the glass transition temperature in the rubber state of the polymer is higher compared to the glassy state below T_g_. Based on the experimental data of this study, the prediction of diffusion coefficients can therefore be considered over-estimative. The diffusion coefficients for Tritan were determined to be below T_g_, which is also the typical temperature range for its food contact applications. In addition, food simulants might swell the surface of the polymers and might increase migration [11]. The diffusion coefficients in this study were determined without any swelling effects. As food typically does not swell the packaging, these are therefore the realistic diffusion coefficients. Aggressive food simulants like 50% or 95% ethanol, however, might increase migration for both polymers, which is not considered in the prediction parameters for the diffusion coefficients.

For PEF, the monomer furan dicarboxylic acid is authorized to be used in combination with ethylene glycol. In the safety evaluation, the EFSA concluded that PEF does not raise a safety concern if the migration of all oligomers less than 1000 g/mol does not exceed a migration limit of 50 µg/kg food [11]. The results of this study show that this limit value can hardly be exceeded as the concentration of oligomers in PEF would have to be very high. However, due to lacking quantitative data on the concentration of oligomers in PEF, this still needs to be verified. The approach used in this study is suitable for testing the compliance of PEF packaging. Regarding Tritan, quantitative data are rare in the scientific literature on the concentration of substances in the polymer relevant for migration and than on migration into food simulants. The low diffusive behaviour of Tritan determined in this study is in good agreement with the experimental results mentioned in the scientific literature. Quantitative data on the concentrations of NIAS in Tritan should be determined in order to confirm compliance with the legal migration limits.

The results of this study clearly show that the activation energy has a huge influence on migration. The activation energy of diffusion determines how the diffusion coefficient increases when the temperature is increased. The four polyesters—PEF, PEN, PET, and Tritan—behave differently here. PEF shows the greatest increase in activation energy with the increase in the size of the molecule, followed by PEN, PET, and then Tritan. This different increase mean that the diffusion behaviour of the four investigated polyesters can be reversed at different temperatures. At room temperature and up to 70 °C, PEF is the least diffusive polyester. At 100 °C, however, PEN becomes the least diffusive polyester. The results show that knowledge of the activation energies is important for predicting migration. Setting the activation energies for all molecules to 80 kJ/mol or 100 kJ/mol, as the currently recognized prediction model does [20,21], can lead to incorrect results.

## Figures and Tables

**Table 1:** Results for the activation energies of diffusion E_A_ and the pre-exponential factor D_0_ for PEF.

Substance	Molecular Volume [Å^3^]	Activation Energy E_A_ [kJ/mol]	Pre-Exponential Factor D_0_ [cm^2^/s]	Temperature Range, Number of Kinetic Points	Correlation Coefficient r^2^ of the Arrhenius Plot
methane	26.64	24.5	2.82 × 10^−7^	90–120 °C, 6 points	0.5043
ethane	45.76	77.9	3.25 × 10^0^	90–120 °C, 6 points	0.9206
methanol	37.21	18.6	6.73 × 10^−8^	100–120 °C, 5 points	0.3751
ethanol	54.02	105.8	1.41 × 10^4^	100–120 °C, 5 points	0.9933
acetone	64.74	184.2	3.49 × 10^14^	100–120 °C, 5 points	0.9941
*n*-propane	62.56	176.6	2.25 × 10^13^	100–120 °C, 5 points	0.9847
1-propanol	70.82	182.6	9.71 × 10^13^	100–120 °C, 5 points	0.9958
2-butanone	81.54	222.0	2.20 × 10^19^	100–120 °C, 5 points	0.9949
1-butanol	87.62	222.9	1.38 × 10^19^	100–120 °C, 5 points	0.9912
ethyl acetate	90.53	211.8	7.78 × 10^17^	100–120 °C, 5 points	0.9777
*n*-pentane	96.16	253.6	1.92 × 10^23^	105–120 °C, 4 points	0.9716
2-pentanone	98.34	243.5	8.82 × 10^21^	100–120 °C, 5 points	0.9967
1-pentanol	104.42	263.3	2.46 × 10^24^	105–120 °C, 4 points	0.9867
*n*-hexane	113.0	245.5	9.78 × 10^21^	105–120 °C, 4 points	0.9857
hexanal	115.1	256.3	2.83 × 10^23^	105–120 °C, 4 points	0.9812
2-hexanone	115.2	259.1	7.49 × 10^23^	100–120 °C, 5 points	0.9973
*n*-butyl acetate	124.1	257.7	4.06 × 10^23^	105–120 °C, 4 points	0.9761
*n*-heptane	129.8	266.4	4.42 × 10^24^	105–120 °C, 4 points	0.9858
2-heptanone	132.0	264.3	2.77 × 10^24^	100–120 °C, 5 points	0.9962
*n*-octane	146.6	276.4	7.09 × 10^25^	105–120 °C, 4 points	0.9871
2-octanone	148.8	266.0	3.83 × 10^24^	105–120 °C, 4 points	0.9937

**Table 2:** Results for the activation energies of diffusion E_A_ and the pre-exponential factor D_0_ for Tritan.

Substance	Molecular Volume [Å^3^]	Activation Energy E_A_ [kJ/mol]	Pre-Exponential Factor D_0_ [cm^2^/s]	Temperature Range, Number of Kinetic Points	Correlation Coefficient r^2^ of the Arrhenius Plot
methane	26.64	15.5	3.43 × 10^−7^	50–80 °C, 4 points	0.8527
ethane	45.76	47.4	1.51 × 10^−2^	50–80 °C, 4 points	0.9961
ethanol	54.02	54.7	8.73 × 10^−2^	80–100 °C, 5 points	0.9745
acetone	64.74	70.1	9.07	50–100 °C, 6 points	0.9973
*n*-propane	62.56	73.3	2.14 × 10^1^	50–80 °C, 4 points	0.9999
1-propanol	70.82	76.1	2.58 × 10^1^	80–100 °C, 5 points	0.9947
*n*-butane	79.36	82.1	1.36 × 10^2^	50–80 °C, 4 points	0.9987
2-butanone	81.54	77.3	4.16 × 10^1^	50–100 °C, 6 points	0.9973
1-butanol	87.62	85.2	2.11 × 10^2^	80–100 °C, 5 points	0.9950
*n*-pentane	96.16	95.4	5.36 × 10^3^	70–90 °C, 5 points	0.9951
2-pentanone	98.34	86.1	2.70 × 10^2^	50–100 °C, 6 points	0.9964
1-pentanol	104.4	92.9	1.17 × 10^3^	80–100 °C, 5 points	0.9899
*n*-hexane	113.0	92.9	9.65 × 10^2^	70–90 °C, 5 points	0.9738
2-hexanone	115.2	94.6	2.11 × 10^3^	50–100 °C, 6 points	0.9944
1-hexanol	121.2	102.5	1.28 × 10^4^	80–100 °C, 5 points	0.9715
*n*-heptane	129.8	98.4	2.82 × 10^3^	70–90 °C, 5 points	0.9788
2-heptanone	132.0	104.1	2.37 × 10^4^	70–100 °C, 4 points	0.9810
1-heptanol	138.0	119.8	1.87 × 10^6^	80–100 °C, 5 points	0.9663
*n*-octane	146.6	114.4	2.94 × 10^5^	75–90 °C, 4 points	0.9927

**Table 3:** Modelling parameters a to d for PEF and Tritan.

Parameter	PEF	Tritan
a [1/K]	2.46 × 10^−3^	2.20 × 10^−3^
b [cm^2^/s]	3.09 × 10^−10^	3.14 × 10^−8^
c [Å^3^]	27.9	21.0
d [1/K]	4.54 × 10^−5^	1.41 × 10^−4^

## Data Availability

Data is contained within the article or Supplementary Material.

## Supplementary Materials

The following supporting information can be downloaded at https://www.mdpi.com/article/10.3390/polym17192674/s1: Tables S1 and S2—Diffusion coefficients for PEF and Tritan. Figures S1 and S2—Correlation of diffusion coefficients with molecular volume compared to predicted D_P_ for PEF and Tritan.
