# Supplementary material for: Diffusion Behavior of Polyethylene Furanoate (PEF) and Tritan as Sustainable Polyester Packaging Materials
Source: Polymers (Basel). 2025 Oct 2;17(19):2674. doi: 10.3390/polym17192674 (PMC12526604; doi:10.3390/polym17192674)
Supplement: Supplementary file 1 [file polymers-17-02674-s001.zip › polymers-3861509-supplementary.pdf]

polymers

MDPI

Supplementary Data

Diffusion behavior of polyethylene furaonate (PEF) and Tritan as sustainable polyester packaging materials

Frank Welle [1,*]

[1] Fraunhofer Institute for Process Engineering and Packaging (IVV), Giggenhauser Straße 35, 85354 Freising, Germany
* Correspondence: frank.welle@ivv.fraunhofer.de

Table S1 and S2 contain the diffusion coefficients for PEF and Tritan. Figure S1 and S2 show correlation of diffusion coefficients with molecular volume compared to predicted D_P for PEF and Tritan.

Table S1. Diffusion coefficients, activation energies of diffusion and the pre-exponential factor for PEF

Substance	Molecular weight, g/mol	Molecular Volume, Å³	Temperature, °C	Diffusion coefficient D_P, cm²/s	Activation energy E_A, kJ/mol	Pre-exponential factor D_0, cm²/s
methane	16.0	28.64	90	1.18×10^{-10}	24.5	2.82×10^{-7}
			95	7.79×10^{-11}		
			100	1.14×10^{-10}		
			105	1.28×10^{-10}		
			110	1.76×10^{-10}		
			115	1.27×10^{-10}		
			120	1.38×10^{-10}		
ethane	30.1	45.76	90	1.16×10^{-10}	77.9	3.25×10^{0}
			95	2.47×10^{-11}		
			100	4.09×10^{-11}		
			105	6.47×10^{-11}		
			110	1.05×10^{-10}		
			115	1.01×10^{-10}		
			120	1.24×10^{-10}		
methanol	32.0	37.21	100	1.56×10^{-10}	18.6	6.73×10^{-8}
			105	1.84×10^{-10}		
			110	2.65×10^{-10}		
			115	1.83×10^{-10}		
			120	2.28×10^{-10}		
n-propane	44.1	62.56	100	3.67×10^{-12}	176.6	2.25×10^{13}
			105	9.40×10^{-12}		
			110	2.31×10^{-11}		
			115	3.77×10^{-11}		
			120	6.80×10^{-11}		
ethanol	46.1	54.02	100	2.12×10^{-11}	105.8	1.41×10^{4}
			105	3.38×10^{-11}		

Substance	Molecular weight, g/mol	Molecular Volume, Å³	Temperature, °C	Diffusion coefficient D_P, cm²/s	Activation energy E_A, kJ/mol	Pre-exponential factor D_0, cm²/s
			110	5.85×10^{-11}		
			115	8.30×10^{-11}		
			120	1.18×10^{-10}		
acetone	58.1	64.74	100	5.74×10^{-12}	184.2	3.49×10^{14}
			105	1.14×10^{-11}		
			110	3.13×10^{-11}		
			115	5.48×10^{-11}		
			120	1.14×10^{-10}		
n-butane	58.1	79.36	115	1.97×10^{-11}		
			120	4.11×10^{-11}		
1-propanol	60.1	70.82	100	2.87×10^{-12}	182.6	9.71×10^{13}
			105	5.35×10^{-12}		
			110	1.22×10^{-11}		
			115	2.89×10^{-11}		
			120	5.21×10^{-11}		
2-butanone	72.1	81.54	100	1.88×10^{-12}	222.0	2.20×10^{19}
			105	4.17×10^{-12}		
			110	1.38×10^{-11}		
			115	2.74×10^{-11}		
			120	6.49×10^{-11}		
n-pentane	72.2	96.20	105	1.50×10^{-12}	253.6	1.92×10^{23}
			110	5.94×10^{-12}		
			115	1.81×10^{-11}		
			120	3.14×10^{-11}		
1-butanol	74.1	87.62	100	9.63×10^{-13}	222.9	1.38×10^{19}
			105	1.92×10^{-12}		
			110	5.27×10^{-12}		
			115	1.66×10^{-11}		
			120	3.16×10^{-11}		
2-pentanone	86.1	98.34	100	6.96×10^{-13}	243.5	8.82×10^{21}
			105	1.97×10^{-12}		
			110	6.61×10^{-12}		
			115	1.45×10^{-11}		
			120	3.76×10^{-11}		
n-hexane	86.2	112.96	105	1.09×10^{-12}	245.5	9.78×10^{21}
			110	3.47×10^{-12}		
			115	1.09×10^{-11}		
			120	2.03×10^{-11}		
ethylacetate	88.1	90.53	100	1.56×10^{-12}	211.8	7.78×10^{17}
			105	4.32×10^{-12}		
			110	1.11×10^{-11}		
			115	3.27×10^{-11}		
			120	4.32×10^{-11}		
1-pentanol	88.2	104.42	105	9.76×10^{-13}	263.3	2.46×10^{24}
			110	3.10×10^{-12}		
			115	1.13×10^{-11}		
			120	2.20×10^{-11}		
cis-hexenol	100.2	115.04	110	1.90×10^{-12}		

Substance	Molecular weight, g/mol	Molecular Volume, Å^3	Temperature, °C	Diffusion coefficient D_P, cm^2/s	Activation energy E_A, kJ/mol	Pre-exponential factor D_0, cm^2/s
			115	5.08×10^{-12}		
			120	1.24×10^{-11}		
2-hexanone	100.2	115.15	100	3.84×10^{-13}	259.1	7.49×10^{23}
			105	1.16×10^{-12}		
			110	4.13×10^{-12}		
			115	9.78×10^{-12}		
			120	2.67×10^{-11}		
hexanal	100.2	115.39	105	9.53×10^{-13}	256.3	2.83×10^{23}
			110	3.62×10^{-12}		
			115	1.09×10^{-11}		
			120	2.08×10^{-11}		
n-heptane	100.2	129.77	105	6.33×10^{-13}	266.4	4.42×10^{24}
			110	2.21×10^{-12}		
			115	7.69×10^{-12}		
			120	1.51×10^{-11}		
1-hexanol	102.2	121.22	110	1.67×10^{-12}		
			115	8.02×10^{-12}		
			120	1.61×10^{-11}		
2-heptanone	114.2	131.95	100	2.67×10^{-13}	264.3	2.77×10^{24}
			105	8.25×10^{-13}		
			110	3.13×10^{-12}		
			115	7.39×10^{-12}		
			120	2.00×10^{-11}		
n-octane	114.2	146.57	105	4.37×10^{-13}	276.4	7.09×10^{25}
			110	1.51×10^{-12}		
			115	5.71×10^{-12}		
			120	1.16×10^{-11}		
n-butylacetate	116.2	124.13	105	8.78×10^{-13}	257.7	4.06×10^{23}
			110	3.60×10^{-12}		
			115	1.05×10^{-11}		
			120	1.97×10^{-11}		
1-heptanol	116.2	138.03	110	1.20×10^{-12}		
			115	6.18×10^{-12}		
			120	1.25×10^{-11}		
2-octanone	128.2	148.75	105	6.21×10^{-13}	266.0	3.83×10^{24}
			110	2.42×10^{-12}		
			115	5.93×10^{-12}		
			120	1.66×10^{-11}		
n-nonane	128.3	163.37	110	1.07×10^{-12}		
			115	4.52×10^{-12}		
			120	9.42×10^{-12}		
isoamylacetate	130.2	140.72	110	3.57×10^{-12}		
			115	1.06×10^{-11}		
			120	2.03×10^{-11}		
1-octanol	130.2	154.83	110	1.81×10^{-12}		
			115	5.14×10^{-12}		
			120	1.05×10^{-11}		
limonene	136.2	157.30	110	1.12×10^{-12}		

Substance	Molecular weight, g/mol	Molecular Volume, Å³	Temperature, °C	Diffusion coefficient D_P, cm²/s	Activation energy E_A, kJ/mol	Pre-exponential factor D_0, cm²/s
			115	3.08×10^{-12}		
			120	8.67×10^{-12}		
n-decane	142.3	180.17	110	1.07×10^{-12}		
			115	3.83×10^{-12}		
			120	7.95×10^{-12}		
methylsalicylate	152.2	136.59	110	2.01×10^{-12}		
			115	5.38×10^{-12}		
			120	1.27×10^{-11}		
n-undecane	156.3	196.97	110	7.11×10^{-13}		
			115	3.29×10^{-12}		
			120	6.32×10^{-12}		
eugenol	164.2	162.14	110	8.84×10^{-13}		
			115	2.48×10^{-12}		
			120	7.30×10^{-12}		
diphenyloxide	170.2	164.44	110	1.22×10^{-12}		
			115	3.42×10^{-12}		
			120	9.79×10^{-12}		
n-dodecane	170.3	213.78	110	5.37×10^{-13}		
			115	2.81×10^{-12}		
			120	6.47×10^{-12}		
n-tridecane	184.4	230.58	115	2.48×10^{-12}		
			120	5.97×10^{-12}		
n-tetradecane	198.4	247.38	115	2.05×10^{-12}		
			120	5.57×10^{-12}		
n-pentadecane	212.4	264.18	115	2.09×10^{-12}		
			120	5.18×10^{-12}		
n-hexadecane	226.5	280.98	115	2.50×10^{-12}		
			120	5.15×10^{-12}		

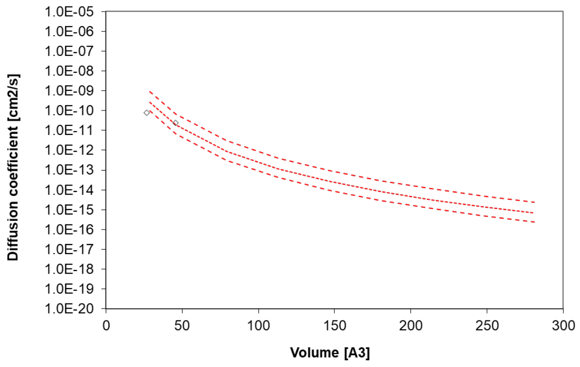

95 °C

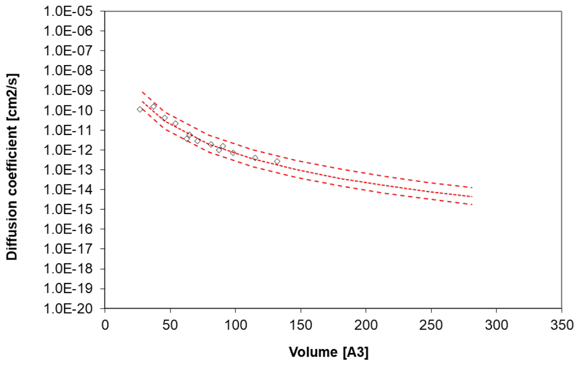

100 °C

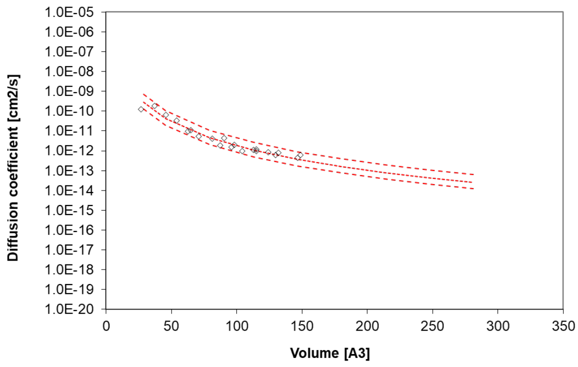

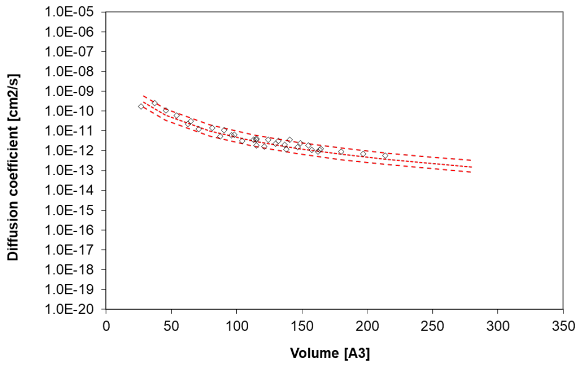

105 °C **110 °C**

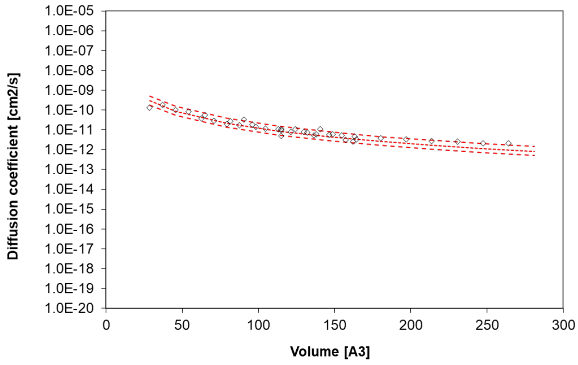

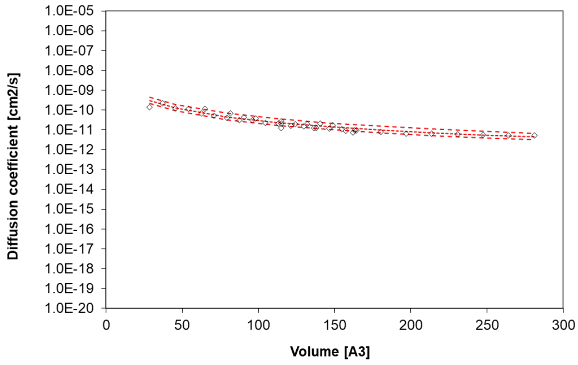

115 °C **120 °C**

Figure S1. Correlation between the diffusion coefficients in PEF and the molecular volume at temperatures between 95 °C and 120 °C. Solid line: predicted from Equation 3 with parameters from Table 3, dashed lines ±20% in volume

Table S2. Diffusion coefficients, activation energies of diffusion and the pre-exponential factor for Tritan

Substance	Molecular weight, g/mol	Molecular Volume, Å³	Temperature, °C	Diffusion coefficient D_P, cm²/s	Activation energy E_A, kJ/mol	Pre-exponential factor D_0, cm²/s
methane	16.0	27.90	50	1.48×10^{-9}	15.5	3.43×10^{-7}
			60	1.44×10^{-9}		
			70	1.98×10^{-9}		
			80	2.30×10^{-9}		
ethane	30.1	45.76	50	3.32×10^{-10}	47.4	1.51×10^{-2}
			60	5.35×10^{-10}		
			70	9.67×10^{-10}		
			80	1.44×10^{-9}		
methanol	32.0	37.21	90	4.27×10^{-9}		
			95	4.92×10^{-9}		
			100	4.18×10^{-9}		
n-propane	44.1	62.56	50	3.03×10^{-11}	73.3	2.14×10^{1}
			60	6.83×10^{-11}		
			70	1.50×10^{-10}		
			80	3.06×10^{-10}		
ethanol	46.1	54.02	80	6.48×10^{-10}	54.7	8.73×10^{-2}

Substance	Molecular weight, g/mol	Molecular Volume, Å^3	Temperature, °C	Diffusion coefficient D_P, cm^2/s	Activation energy E_A, kJ/mol	Pre-exponential factor D_0, cm^2/s
			85	9.94×10^{-10}		
			90	1.21×10^{-9}		
			95	1.49×10^{-9}		
			100	1.84×10^{-9}		
acetone	58.1	64.74	50	4.09×10^{-11}	70.1	9.07
			60	8.57×10^{-11}		
			70	2.02×10^{-10}		
			80	4.26×10^{-10}		
			90	7.20×10^{-10}		
			100	1.30×10^{-9}		
n-butane	58.1	79.36	50	7.57×10^{-12}	82.1	1.36×10^{2}
			60	1.83×10^{-11}		
			70	4.17×10^{-11}		
			80	1.03×10^{-10}		
1-propanol	60.1	70.82	80	1.48×10^{-10}	76.1	2.58×10^{1}
			85	1.99×10^{-10}		
			90	2.86×10^{-10}		
			95	3.93×10^{-10}		
			100	5.99×10^{-10}		
2-butanone	72.1	81.54	50	1.36×10^{-11}	77.3	4.16×10^{1}
			60	2.92×10^{-11}		
			70	7.27×10^{-11}		
			80	1.69×10^{-10}		
			90	2.85×10^{-10}		
			100	6.43×10^{-10}		
n-pentane	72.2	96.20	70	1.66×10^{-11}	95.4	5.36×10^{3}
			75	2.53×10^{-11}		
			80	3.95×10^{-11}		
			85	6.12×10^{-11}		
			90	1.07×10^{-10}		
1-butanol	74.1	87.62	80	5.37×10^{-11}	85.2	2.11×10^{2}
			85	7.75×10^{-11}		
			90	1.13×10^{-10}		
			95	1.61×10^{-10}		
			100	2.61×10^{-10}		
2-pentanone	86.1	98.34	50	3.40×10^{-12}	86.1	2.70×10^{2}
			60	8.01×10^{-12}		
			70	2.12×10^{-11}		
			80	5.41×10^{-11}		
			90	9.47×10^{-11}		
			100	2.61×10^{-10}		
n-hexane	86.2	112.96	70	8.09×10^{-12}	92.9	9.65×10^{2}
			75	1.03×10^{-11}		
			80	1.58×10^{-11}		
			85	2.67×10^{-11}		
			90	4.75×10^{-11}		
ethylacetate	88.1	90.53	80	1.49×10^{-10}		
			90	2.65×10^{-10}		

Substance	Molecular weight, g/mol	Molecular Volume, Å^3	Temperature, °C	Diffusion coefficient D_P, cm^2/s	Activation energy E_A, kJ/mol	Pre-exponential factor D_0, cm^2/s
			100	4.10×10^{-10}		
1-pentanol	88.2	104.42	80	2.17×10^{-11}	92.9	1.17×10^{3}
			85	3.50×10^{-11}		
			90	5.00×10^{-11}		
			95	7.10×10^{-11}		
			100	1.27×10^{-10}		
2-hexanone	100.2	115.15	50	1.14×10^{-12}	94.6	2.11×10^{3}
			60	2.85×10^{-12}		
			70	8.25×10^{-12}		
			80	2.41×10^{-11}		
			90	4.17×10^{-11}		
			100	1.36×10^{-10}		
cis-hexenol	100.2	115.04	90	3.91×10^{-11}		
			100	3.74×10^{-11}		
hexanal	100.2	115.39	80	2.02×10^{-11}		
			90	4.09×10^{-11}		
			100	1.23×10^{-10}		
n-heptane	100.2	129.77	70	3.31×10^{-12}	98.4	2.82×10^{3}
			75	4.63×10^{-12}		
			80	6.96×10^{-12}		
			85	1.18×10^{-11}		
			90	2.24×10^{-11}		
1-hexanol	102.2	121.22	80	8.79×10^{-12}	102.5	1.28×10^{4}
			85	1.61×10^{-11}		
			90	2.05×10^{-11}		
			95	3.17×10^{-11}		
			100	6.52×10^{-11}		
2-heptanone	114.2	131.95	70	3.39×10^{-12}	104.1	2.37×10^{4}
			80	1.04×10^{-11}		
			90	1.94×10^{-11}		
			100	7.21×10^{-11}		
n-octane	114.2	146.57	75	2.10×10^{-12}	114.4	2.94×10^{5}
			80	3.41×10^{-12}		
			85	5.66×10^{-12}		
			90	1.09×10^{-11}		
n-butylacetate	116.2	124.13	80	2.13×10^{-11}		
			90	4.23×10^{-11}		
			100	1.21×10^{-10}		
1-heptanol	116.2	138.03	80	3.54×10^{-12}	119.8	1.87×10^{6}
			85	7.33×10^{-12}		
			90	9.55×10^{-12}		
			95	1.56×10^{-11}		
			100	3.75×10^{-11}		
2-octanone	128.2	148.75	80	4.95×10^{-12}		
			90	9.35×10^{-12}		
			100	4.09×10^{-11}		
n-nonane	128.3	163.37	85	2.25×10^{-12}		
			90	5.04×10^{-12}		

Substance	Molecular weight, g/mol	Molecular Volume, $Å^3$	Temperature, °C	Diffusion coefficient D_P, cm^2/s	Activation energy E_A, kJ/mol	Pre-exponential factor D_0, cm^2/s
isoamylacetate	130.2	140.72	80	1.89×10^{-11}		
			100	1.17×10^{-10}		
1-octanol	130.2	154.83	85	3.26×10^{-12}		
			95	8.58×10^{-12}		
			100	2.07×10^{-11}		
limonene	136.2	157.30	90	6.59×10^{-12}		
			100	2.14×10^{-11}		
nonanal	142.2	165.79	90	5.38×10^{-12}		
			100	3.33×10^{-11}		
n-decane	142.3	180.17	90	2.79×10^{-12}		
methylsalicylate	152.2	136.59	80	1.01×10^{-11}		
			90	2.36×10^{-11}		
			100	7.96×10^{-11}		
menthol	156.3	177.21	90	5.37×10^{-12}		
citronellol	156.3	181.79	100	9.87×10^{-12}		
eugenol	164.2	162.14	90	2.99×10^{-12}		
			100	2.25×10^{-11}		
diphenyloxide	170.2	164.44	90	4.80×10^{-12}		
			100	2.73×10^{-11}		
linalylacetate	196.3	212.10	100	1.48×10^{-11}		

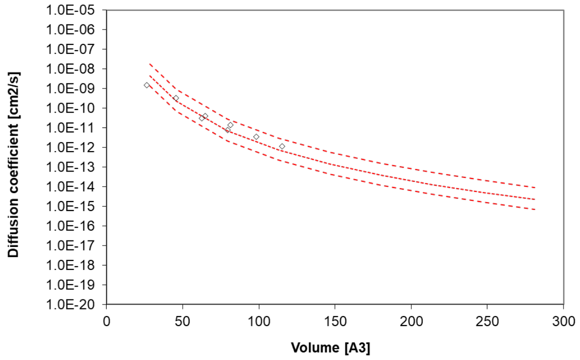

50 °C

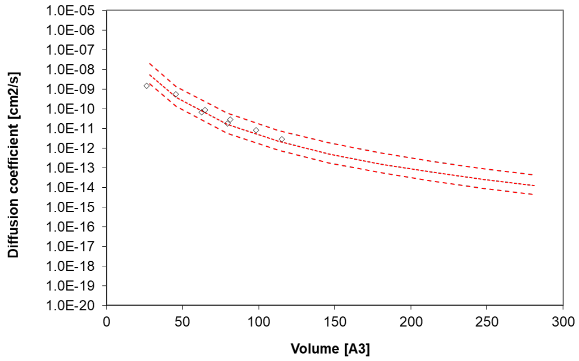

60 °C

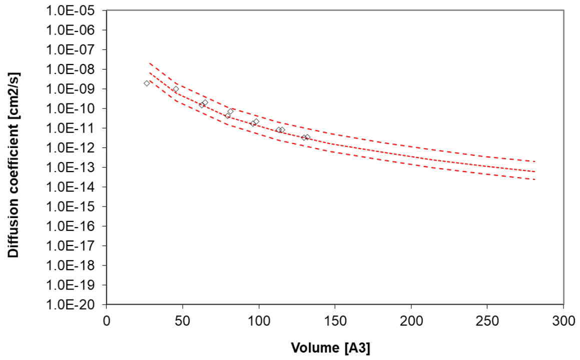

70 °C

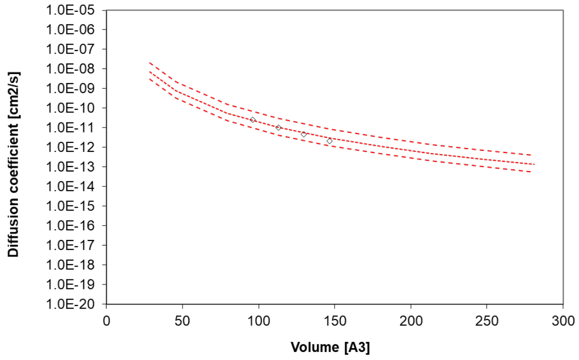

75 °C

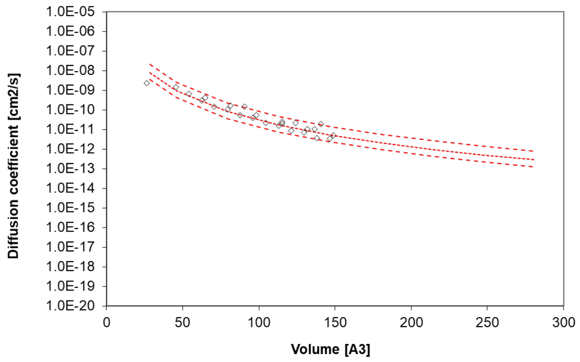

80 °C

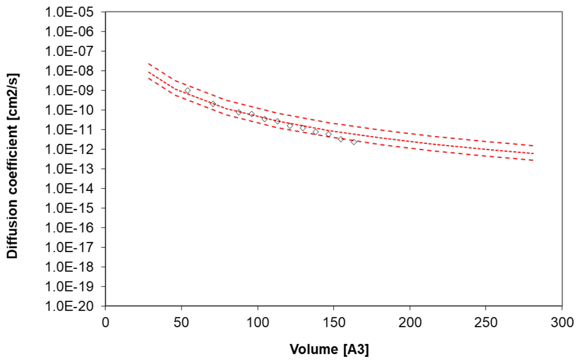

85 °C

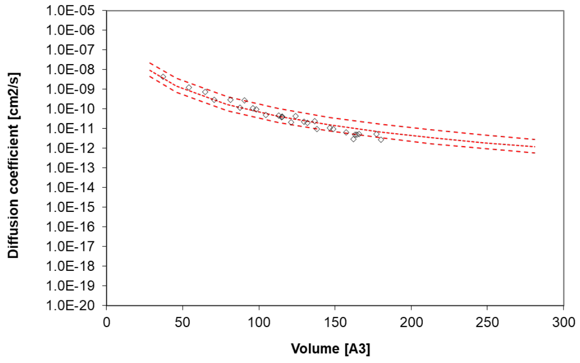

90 °C

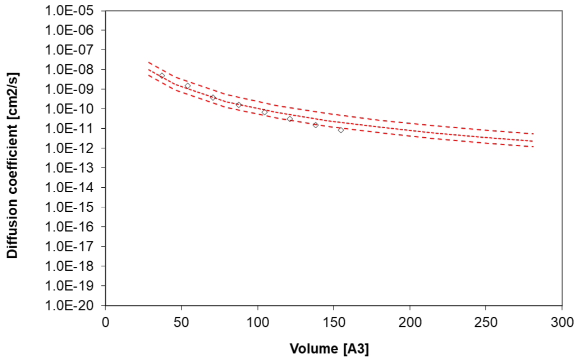

95 °C

Figure S2. Correlation between the diffusion coefficients in Tritan and the molecular volume at temperatures between 95 °C and 120 °C. Solid line: predicted from Equation 3 with parameters from Table 3, dashed lines ±20% in volume
